# Dr. Henry Maudsley

**Published:** 1918-02-23

**Authors:** 


### DR. HENRY MAUDSLEY

### (born 1835, died January 23, 1918).

A correspondent has sent us an interesting statement on the work of Dr. Henry Maudsley, from which we feel we should find room for the following:—

Maudsley's studies—embodied in such works as the "Physiology and Pathology of Mind," "Body and Mind," "Life in Mind and Conduct"—led to the conclusion that it is not possible to separate any processes in the human body from the general scheme of Nature. His last volume, "Organic to Human," published in 1917, emphasises this, and renders it clear also that mature deliberation had not caused him to depart from the views enunciated previously by him during half a century. The study of psychology and of psychiatry cannot, however, be kept within the limited bounds which are too frequently assigned to them. Maudsley has clearly demonstrated this; and he has shown that problems in, for example, philosophy, literature, history, and sociology become easier of solution when the results derived from the study of normal and morbid mental processes are applied to them. In a word, his outlook upon life was not restricted to a narrow and specialist sphere. This is particularly obvious in "Organic to Human," which may be said to summarise the thought of a lifetime. In it he applies the biological method to social organisation, as well as to psychology, with conspicuous clearness and success.

In philosophy he has been one of the most doughty advocates of materialism as opposed to spiritualism or vitalism. It is a doctrine without any particular emotional appeal, and it has not, therefore, made converts rapidly; while it is, for the most part, treated almost contemptuously by many who have not taken the trouble to study intimately the structures through which, as they are now practically compelled to admit, their hypothetical "mind" acts. The mechanistic conception, too, almost inevitably leads to pessimism—that is to say, it shatters many of our cherished illusions—and this is, therefore, another factor which makes for unpopularity. But though it may, for example, make us despair of human perfectibility or even of rapid amelioration of social conditions, it does not preclude steady progress once we have realised that what we have to aim at is the steady evolution of mind by means of "adaptive working experience and its consequent physical structuralisation in the brain—the literal in-struction or in-formation, that is, of cerebral plexuses of structure and function."

He gave practical expression to his desire to co-ordinate the study of mental disorders with medical research in general in the munificent gift which has resulted in the building of the Maudsley Hospital. The main object, however, which he had in view in the foundation of this institution was to provide for the early treatment of cases of acute insanity in order to obviate the necessity of these unfortunate individuals being sent at once to asylums. Whatever views may be held as to the possibility of Maudsley's ideas in this respect being carried out successfully in practice, a generous meed of praise is due to the philanthropic spirit which inspired the gift.

